# Systematic Configurator for Complexity Management in Manufacturing Systems

**DOI:** 10.3390/e26090747

**Published:** 2024-08-31

**Authors:** Germán Herrera-Vidal, Jairo R. Coronado-Hernández, Breezy P. Martínez Paredes, Blas Oscar Sánchez Ramos, David Martinez Sierra

**Affiliations:** 1 Industrial Engineering School, Universidad del Sinú, Cartagena 130001, Colombia; 2 Department of Productivity and Innovation, Universidad de la Costa, Barranquilla 080016, Colombia; 3 Faculty of Engineering, Universidad Nacional Mayor de San Marcos, Lima 15081, Peru; 4 Faculty of Engineering, Universidad Nacional Autónoma de Tayacaja Daniel Hernández Morillo, Pampas 09156, Peru; oscarsanchez@unat.edu.pe; 5 Faculty of Engineering, Universidad Simón Bolívar, Barranquilla 080005, Colombia; dmartinez@unisimonbolivar.edu.co

**Keywords:** complexity, entropic, configurator, management, manufacturing, system

## Abstract

Complexity management in manufacturing systems is crucial for the economic growth of countries, as efficient management can significantly improve business performance and ensure competitiveness in globalized markets. This research aims to develop a complexity management configurator that identifies critical effects, proposes solution strategies, and optimizes processes through a Lean Production and Industry 4.0 approach. To this end, its systematic design integrates the key stages of management: planning, organization, management and control. The research was structured as an applied study, implementing three main panels: general information, application of a diagnostic tool at each stage of the administrative process, and results focused on measuring the complexity and implementation of advanced technological solutions. The tool enables manufacturing companies not only to diagnose sources of complexity, but also to optimize their operations by adopting Lean methodologies and Industry 4.0 technologies. The findings show how the integration of these strategies contributes to reducing both static and dynamic complexity, resulting in greater operational efficiency and improved performance in an increasingly competitive industrial environment. In conclusion, the proposed configurator is positioned as a key tool to improve the competitiveness and sustainability of manufacturing companies by offering a comprehensive approach to complexity management that is tailored to the demands of modern industry.

## 1. Introduction

The economic growth of countries depends on an adequate management of complexity in manufacturing systems [1,2], as this is important to direct strategies towards a better corporate performance. Studies on complexity are becoming more notorious and frequent [3,4], because decision makers seek to manage it, in order to have a competitive impact in complex and globalized markets [5]. According to [6], complexity depends on its temporal variability, due to industry and company specific effects, without leaving aside the components that will interact in the development of manufacturing [7]. Complexity increases when the system faces a series of strategies nested with the internal and external environment of the company [8].

According to [9], an increase in complexity in manufacturing systems negatively affects all aspects of manufacturing including operational, tactical and strategic. They are also linked to the occurrence of machine failures, quality problems, material failures and people absenteeism [10]. In this context, the authors of Ref. [11] believe that a company must manage the complexity of its system in order to keep operational costs low. The studies of [12] state that 25% of the total costs of manufacturing companies are due to the complexity within the process and the different characteristics associated with the product. This makes it necessary to have methods and tools for proper management [13] and correct application of strategies for analysis and understanding [14].

Several methods for managing complexity have been developed in the literature, from aerospace domain [15], in mechatronic systems [16,17], in automotive design [18], in the context of project management [19,20,21,22,23], in circular economy [24], and to the construction sector [25,26]. However, these methods have not yet been fully adopted by the manufacturing systems’ engineering community [27]. Moreover, the industrial methods used today to assess complexity are not accurate, as they are largely subjective and non-systemic [28]. According to [29], current manufacturing is still based on traditional methods and can hardly cope with the increasing complexity of systems.

Complexity in manufacturing systems can be addressed from various perspectives, as evidenced by recent studies in this field. For example, Ref. [30] proposes a three-step approach for operational planning in complex manufacturing systems, which highlights the need for decision support tools that integrate multiple variables and dynamics of the production process. On the other hand, Ref. [31] presents a hybrid digital model that enables the continuous evolution of large manufacturing systems, which is crucial to adapt to market changes and customization demands. In addition, research by [32] on sustainable production planning using predictive model control demonstrates how complexity can be managed to achieve sustainability goals by integrating advanced modeling techniques. Finally, the digital twin approach to short-term production planning optimization, as discussed in the work of [33], illustrates how emerging technologies can address complexity through simulation and real-time analytics. Taken together, this research underscores that complexity in manufacturing systems is not only a challenge, but also an opportunity to innovate and improve operational efficiency.

From another internal perspective in manufacturing systems, the scientific and academic community has paid more attention to methodologies and models related to the configuration, structure and variation by products and processes [34,35,36,37,38,39,40,41,42], leaving aside the complexity from an administrative or organizational scope [43].

Managing complexity in manufacturing systems presents particular challenges in different geographical contexts. In developing countries, such as Latin America, the adoption of Industry 4.0 technologies to address complexity is limited by barriers such as lack of infrastructure, limited access to capital and the skills gap. Ref. [44] points out that in this region there is a growing interest in models that allow reducing complexity in operations, product deterioration and labor accidents, efficiently using 4.0 technologies. On the other hand, in developed countries, highly complex manufacturing, characterized by long cycle times, unique production paths and high cross-functional coordination, is common in sectors such as aerospace. A report by [45] highlights that these manufacturers face the challenge of rapidly ramping up production to record levels, with more complex products and a less experienced workforce. In Europe, the European Union has prioritized the digitalization of industry through initiatives such as Industry 4.0. Ref. [46] points out that the ability to adopt advanced technologies depends on the degree of standardization and automation of production processes, as well as the ability of companies to successfully implement monitoring, optimization and autonomous management initiatives. In summary, while managing complexity in manufacturing systems is a global challenge, approaches and barriers vary by geographic context. Effectively addressing complexity will require strategies tailored to the specific needs and capabilities of each region.

The main contributions of this work are the following:

(1) To present a method for complexity management in manufacturing systems, which allows the identification of the effects, solution strategies and improvement methods from a Lean Production and Industry 4.0 approach.
(2) To provide a systematic configurator that allows the measurement of complexity in the different administrative stages, consistent with modern methods.
(3) To provide a new mechanism for measuring complexity in an entropic way for manufacturing systems, based on questionnaire-type instruments.

The remainder of the paper is organized as follows. Section 2 provides a review of the literature. Section 3 describes and proposes the methods and materials. Section 4 presents the description and characteristics of the configurator. Section 5 details the results obtained. Section 6 is a discussion of the findings and the results obtained. Section 7 presents the conclusions and possible future studies.

## 2. Literature Review

The meaning of the term “complexity” consulted in the Cambridge Dictionary states that something is “complex” when it is difficult to understand or find an answer to because it has many different parts [47]. According to the Oxford Learner’s Dictionary of Academic English [48], complexity is “the state of being made up of many parts; the state of being difficult to understand”, complex is “made up of many different things or parts that are connected; difficult to understand”, and “complicated” is a synonym for “complex”. According to [49,50], complexity is a new science or area of knowledge, which is studied in different scientific areas, acquiring new conceptualizations and developing approaches relative to each of these areas or knowledge. In the literature review there is no universal definition, since complexity is difficult to define precisely. A genesis definition appears in the works of [51] where a complex system is one that has a large number of parts where the relationships are not simple, they are subject to the volume of elements that exist in the system [52], and are proportional to the amount of information necessary for their interaction [53], making it heterogeneous type of environment [54]. According to [55], complexity is the opposite of simplicity. Likewise, Ref. [56] states that complexity refers to large networks of components without central control and simple rules of operation, which give rise to complex collective behavior of information. Similarly, [57] states that it depends on the size of the system, randomness, asymmetry and constraints.

In the literature, Ref. [58] analyzed the term complexity by focusing on the behavior of multiple interrelated components. Similarly, Ref. [59] did the same but focused on instability, variety and degree of interdependence. However, Ref. [60] states that the complexity of a manufacturing system increases not only with the volume of components, parts, pieces or subassemblies, but also with the quantities of tasks, activities or operations, even when materials do not meet specifications of time, quantity and quality, when absenteeism of people or when machines or equipment fail [34]. According to Aram and Noble [61], complexity is the constant struggle between inactivity and disorder.

In the literature, different types of complexity in manufacturing systems are identified. According to [62], depending on the origin, it can be internal and external, where the internal refers to the variables between the flows within the manufacturing, and the external depends on the variables between the agents of the supply chain [63]. From another perspective, [64,65] state that it can be static (structural) and dynamic (operational), where static represents the time-independent characteristics of a manufacturing system and focuses on the types of subsystems and the strength of interconnections [66] and dynamic represents the operational characteristics of the system and involves temporal and random aspects [67]. For adequate management, it is necessary to identify, measure, analyze and control it [36]; for this purpose, there are qualitative methods that depend on the perception of the people involved in the process and quantitative methods based on data, verification and analysis. There are different approaches and methods to measure complexity, among which are nonlinear dynamics, information theory (entropic), hybrid methods, enumeration and questionnaires. Similarly, there are different types of models classified from a conceptual, theoretical and mathematical perspective [36]. According to [40], the measurement of complexity in manufacturing systems is a metric that serves as a parameter to establish improvement plans, in turn determining that systems with high complexity present more problems than systems with low complexity. In recent research, works that stand out are [40,41] who propose a new hybrid and entropic metric considering the current deficiencies and gaps found in the literature.

Complexity control depends on solution strategies and methods. Ref. [68] lists solution techniques for identifying factors involved in system complexity, focused on product and process redesign, implementation of digital platforms, collaborative planning, adoption of new technologies and processes, and process automation. Similarly, Ref. [69] proposes the use of computer and technological resources. More recently, in [34], they list some management methodologies of Lean Manufacturing, compiled from [70,71,72].

This breadth of review provides a solid foundation for understanding complexity in manufacturing systems, highlighting both existing methodologies and relevant technologies. However, it is crucial to recognize the limitations in previous research and how these limitations have influenced the evolution of approaches to managing complexity.

Despite significant advances in understanding complexity, many previous studies have tended to address complexity in a fragmented manner, focusing on specific aspects, such as internal versus external complexity, or static versus dynamic [16,17,18,19,20,21,22]. This segmentation, while useful for detailed analysis, limits the ability to provide a holistic view of how these dimensions interact in a complex manufacturing environment. In addition, much research focuses on identifying and measuring complexity but lacks a robust approach to its effective management and control [23,24]. Traditional metrics often fail to consider the dynamics and nonlinear nature of complex systems, resulting in management approaches that are insufficient to address real challenges in real time.

A critical limitation in the existing literature is the lack of integration between qualitative and quantitative methodologies. Although advanced approaches such as hybrid and entropic models exist [25,26], these have not been widely adopted or validated in a variety of industrial settings, leaving a gap in the practical applicability of these methods. Furthermore, many of the current proposals do not adequately address the interplay between complexity and other key factors, such as sustainability and resilience, which are increasingly important in modern manufacturing.

Therefore, the present research proposes a new configurator that seeks to overcome these limitations by offering a more holistic approach to the identification, measurement, analysis and control of complexity. This configurator not only incorporates state-of-the-art methodologies, but also integrates them in a way that allows for practical and effective application in real industrial environments. By combining qualitative and quantitative methods, the approach provides a more accurate and contextualized assessment of complexity, addressing the shortcomings observed in previous studies.

Furthermore, it is essential to highlight that the research fills critical gaps in the existing literature by providing a framework for complexity management that is adaptable to different industrial contexts, including scenarios with high variability and demand for customization. This framework is particularly relevant in the era of Industry 4.0, where interconnectedness and automation demand new ways of dealing with complexity that go beyond traditional methodologies. The above described becomes a favorable scenario for the development of configured mechanisms that allow the identification, measurement, analysis and control of complexity in manufacturing systems.

## 3. Materials and Methods

**Figure 1:**
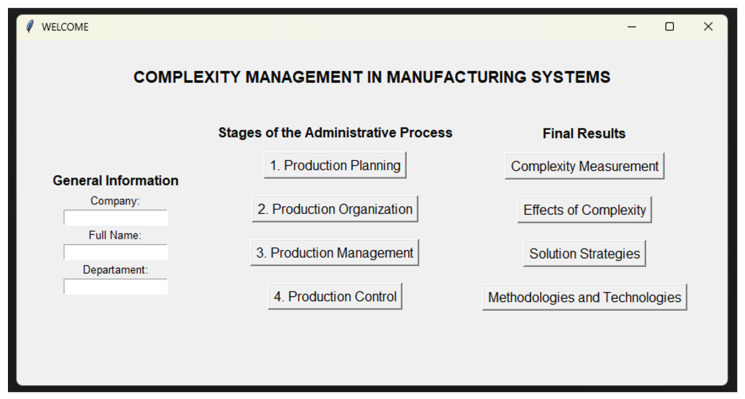
Illustrative visualization of the configurator for complexity management.

The goal of the configurator of development is to enable manufacturing company executives to make accurate decisions to manage complexity tailored to their needs (see Figure 1).

**Figure 2:**
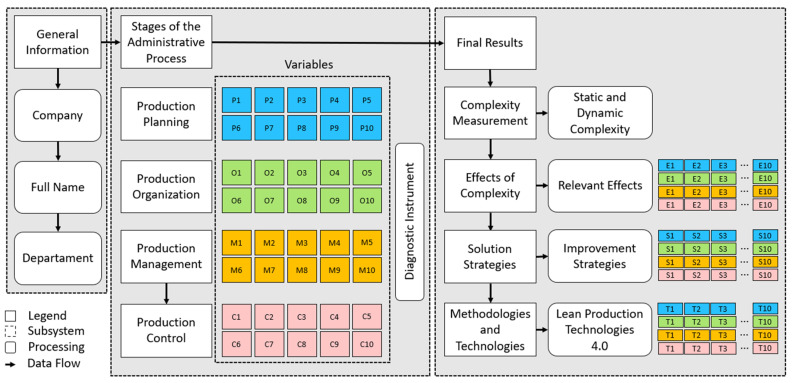
Basic data flows and processing elements. Blue color: Production planning; Green color: Production organization; Orange color: Production management; Pink color: Production control.

The method used during this research consisted of the development of a configurator, composed of three (3) main panels. First, data entered by the user are provided (see Section 4.1). Secondly, the diagnostic instrument applied to obtain information at each stage of the administrative process is filled in (see Section 4.2). Thirdly, a mechanism is proposed for measuring complexity, identifying effects, solution strategies and defining methodologies and modern technologies, the relationship of these elements being vital for an analysis (see Section 4.3). Figure 2 shows a view of the configurator architecture.

## 4. Configurator for Complexity Management

This section offers a complexity management system for manufacturing companies, consisting of diagnostic aspects, measurement, analysis and solution proposals. Methodologically, each of the panels described in the materials and methods section are described.

### 4.1. General Information

The Python programmer is responsible for developing this application of complexity management in manufacturing systems, allowing demonstration of techniques and skills essential for the success of the project. For its implementation, a computer with an 8 GB RAM, 11th Gen Intel(R) Core(TM) i5 processor and a 64-bit operating system running Windows 11 with Python 3.11.4 installed on the system was required. In the first panel of the welcome window, the tool allows the user to fill in the fields in an established order, avoiding inconsistencies and conflicts. Initially the program requests information regarding the company name, followed by the name of the manager or executive of the company and finally the department to which he/she belongs. This information is stored in a spreadsheet for its registration and later relation with the following panels and elements.

### 4.2. Stages in the Administrative Process

According to [73], production management is the process of planning, organizing, directing and controlling the resources and activities related to the production of goods and services of an organization to achieve its strategic and operational objectives. On the other hand [74], their definition states that it includes capacity planning, inventory management, production scheduling, quality control and other activities aimed at optimizing the efficiency and effectiveness of production operations. According to [75], it involves making decisions about resource allocation, shaping production processes and implementing measures for improvement. The above supports and provides a support in the initial construction of the second panel of the configurator, associated with the stages of the administrative process: (i) Planning [76,77,78,79], (ii) Organization [80,81,82,83], (iii) Management [84,85,86,87], and (iv) Control [88,89,90].

In this panel, a survey-type diagnostic instrument is applied, taking into account several variables identified in the literature (see Table 1).

**Figure 3:**
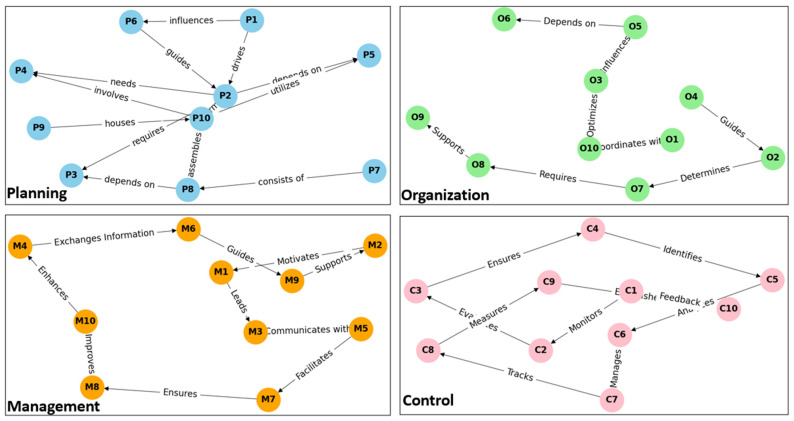
Relationship graph of variables.

The variable selection process begins with the identification of relevant scientific references in each of the stages (see Table 1), ensuring that all of them have a theoretical and practical basis in the industry. Consequently, the interrelationship between them is considered, allowing a holistic analysis. Figure 3 visually shows the connections and relationships between the variables.

**Figure 4:**
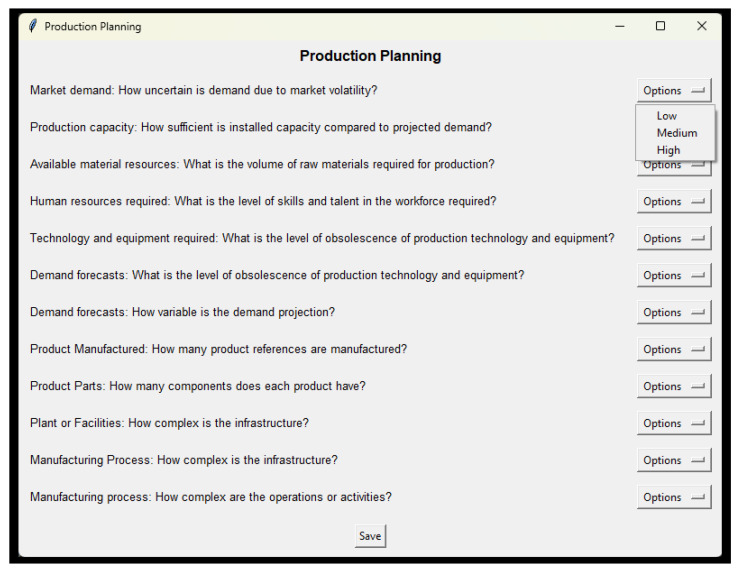
Survey-type instrument for the production planning stage.

Given the above, 40 variables related to the stages of the administrative process were identified. The instrument applied to manage complexity is a questionnaire type, a technique of great interest for researchers to test the impact of complexity in manufacturing systems [131], measure the impact of complexity on business performance [63], investigate the effects of complexity in the supply chain [132], and statistically analyze the effects by productive factors of complexity in manufacturing systems [35]. For its development, a rating scale was applied, consisting of 3 levels (Low-Medium-High), and with scores (1-3-5), respectively (see Figure 4). According to [133], an instrument is valid if it measures what it is supposed to measure, and in a context of implementation in various economic sectors, the reliability and consistency of the application was determined. For this purpose, a group of university academics, SME managers and experts or consultants from the industrial sector were interviewed. The Lawshe (1975) model was systematically used, determined by expert consensus, based on aspects to be evaluated such as structure, relevance and wording [134]. The results of the validity coefficient (CVR) in the 40 declared variables represent a value greater than or equal to 0.75, so that each of the items were approved. Another statistical test developed is the Cronbach’s Alpha consistency analysis, taking into account the results of six samples in subsectors such as metal-mechanic, plastic, chemical, wood, lithographic and food; the statistic is equal to 0.9436, being higher than 0.7, so the instrument is considered reliable and consistent. Similarly, the systematic test–retest method was applied, considering different points in time, where the Pearson correlation coefficient between the scores is equal to 0.8245, reflecting a very high positive correlation, thus assuring the goodness of the instrument’s results.

### 4.3. Final Results

This panel comprises four (4) important aspects that provide results and support decision making, it is worth mentioning that they should be developed sequentially: (i) Complexity measurement; (ii) Complexity effects; (iii) Solution strategies and (iv) Methodologies and technologies.

#### 4.3.1. Complexity Measurement

Shannon’s entropy, developed for information theory, measures the uncertainty or unpredictability of a system, this metric helps to quantify the complexity of processes, variations in production, and uncertainty in demand and resources [135]. Entropy *H*(*X*) of a random variable *X* representing the states of a process is defined as follows:

(1) $$H\left(X\right)=-\sum_{i=1}^{n}p(x_{i}){log}_{2}p(x_{i})$$

where *p*(*x_i_*) is the probability of outcomes of a system being in state i, (i = 1, … , n), *p*(*xi*) *≥* 0, $\sum_{i=1}^{n}p\left(x_{i}\right)\;$ and log_2_(0) = 0.

Authors such as [136,137,138], have made significant contributions to the field of complexity measurement in manufacturing systems using concepts based on Shannon entropy. Their work includes metrics for a measure of static complexity (Equation (2)) and dynamic complexity (Equation (3)).

(2) $$C_{Static}\left(Cs\right)=-\sum_{i=1}^{M}\sum_{j=1}^{N}P_{ij}{log}_{2}P_{ij}$$

(3) $$C_{Dynamic}\left(Cd\right)=-(1-P)\sum_{i=1}^{M}\sum_{j=1}^{N}P_{ij}{log}_{2}P_{ij}$$

Subsequently, [139] proposes a modified approach for a complexity measure providing an additional element to the formulas for static (Equation (4)) and dynamic complexity (Equation (5)) related to dij which is the value of the deviation from the expected value for resource i in state j.

(4) $$C_{Static}\left(Cs\right)=-\sum_{i=1}^{M}\sum_{j=1}^{N}\left[{log}_{2}P_{ij}\right]d_{ij}P_{ij}$$

(5) $$C_{Dynamic}\left(Cd\right)=-(1-P)\sum_{i=1}^{M}\sum_{j=1}^{N}\left[{log}_{2}P_{ij}\right]d_{ij}P_{ij}$$

Consequently, Ref. [40] proposes a modified and updated approach to measure complexity in manufacturing systems from an entropic viewpoint, an innovative element of the authors’ recent work. They consider three aspects: (i) the determination of levels and class amplitude in a quantitative way depending on the amount of data; (ii) calculation of weights (Wij) depending on the number of intervals; and (iii) assignment of weights taking into account that the farther away from the control state, the greater the deviation and therefore the greater the weight. Given the above, Equations (6) and (7) are formulated, which represent the metrics for the entropic measurement of complexity in manufacturing systems.

(6) $$C_{Static}\left(Cs\right)=-\sum_{i=1}^{M}\sum_{j=1}^{N}\left[{log}_{2}P_{ij}\right]W_{ij}d_{ij}P_{ij}$$

(7) $$C_{Dynamic}\left(Cd\right)=-(1-P)\sum_{i=1}^{M}\sum_{j=1}^{N}\left[{log}_{2}P_{ij}\right]W_{ij}d_{ij}P_{ij}$$

where,

P: Probability in the control state.
(1 − P): Probability in out-of-control state.
Pij: Probability of resource i, i = 1, … , M being in state j, j = 1, … , N.
dij: Absolute deviation from the expected results for the condition.
Wij: Weighting of each interval.
M: Number of resources.
N: Number of possible states.

**Figure 5:**
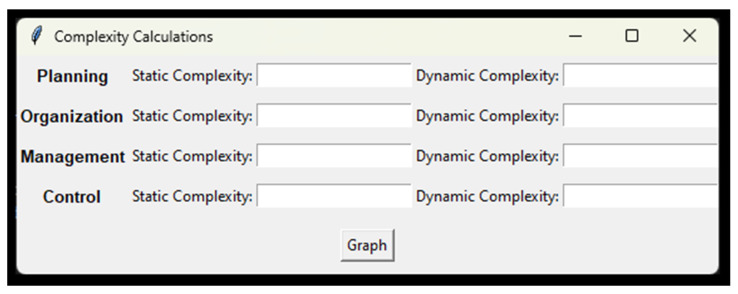
Complexity calculation report window.

The measures for the calculation of complexity in a manufacturing system are measured static (Cs), considering the structure and the degree of difficulty for its management and control, and dynamic (Cd), analyzing its behavior over time. The unit of measurement is given in “bits” which corresponds to the amount of information required to make a binary choice [140]. Finally, the calculations are recorded and saved in an Excel file, which allows the generation of a comparative graph for interpretation and analysis (see Figure 5). This process determines the stage of the administrative process with greater or lesser complexity, providing priority bases for decision making.

#### 4.3.2. Effects of Complexity

To identify the effects of complexity, an interview technique and surveys to qualified and experienced personnel were used, considering the variables and stages identified. The configurator proposes a list composed of forty (40) effects, which are selected according to the selected stage with greater complexity and variables with greater relevance. Table 2 below shows the identification of the effects, which will provide relevant information for the following stages of solution strategies, methodologies and improvement technologies.

#### 4.3.3. Solution Strategies

In order to obtain the effective solution strategies, the brainstorming technique was applied among experts and previous research where similar problems were addressed. Table 3 shows one of the selection windows of the production planning stage, evidencing the different strategies according to the related variable. 

#### 4.3.4. Improvement Methodologies and Technologies

In the resolution of specific problems in manufacturing, modern methodologies focused on lean production are currently distinguished; according to [141], they allow us to achieve operational excellence; and according to [142], can be applicable in various industries. These focus on the elimination of waste, continuous improvement and optimization of production processes. Similarly, advanced digital technologies stand out in manufacturing processes, due to the fourth industrial revolution, where they provide a theoretical framework for the implementation of Industry 4.0 [143] and are today transforming industry and society [144]. The configurator allows the choosing of various methodologies and technologies according to the previously selected effects and solution strategies (see Table 4), supporting decision making and serving as a support for complexity management in manufacturing systems.

### 4.4. Validation of the Configurator

Validation of the configurator is carried out by means of a case study, focused on production management and considering the key administrative stages: planning, organization, management and control. Although the configurator is of an administrative nature, its approach allows the identification of the most complex stage and provides a detailed analysis of the effects, strategies and applicable improvement technologies. In the case study, the configurator was applied to assess the complexity in each of the above-mentioned administrative stages. The assessment included the measurement of specific parameters, such as static and dynamic entropy, which reflect the complexity inherent in each stage. Validation was based on a quantitative analysis of complexity metrics, where the most critical areas were identified, and improvement strategies based on Industry 4.0 technologies were proposed.

Key metrics used for the evaluation included the calculation of static and dynamic entropy, as well as the assessment of redundancy and synergy in the information shared between variables. These metrics allowed a deep understanding of how complexity is distributed throughout the different stages of the production process and how information flows and decision making can be optimized.

### 4.5. Hypothesis

The approach of this hypothesis focuses on how the systematic configurator can optimize both the structure and the operation of a production management system. By intelligently adjusting the distribution of resources and frequencies at each stage of the process, it is possible to reduce static complexity by improving structural stability, while simultaneously minimizing dynamic complexity by increasing responsiveness to fluctuations and instabilities. This balance, achieved through robust management of critical stages, results in a more efficient and resilient production system. The following hypothesis is put forward:

“Implementing a systematic configurator that optimizes resource and frequency allocation, balancing structural stability and operational adaptability, can reduce both static and dynamic complexity at critical production stages, improving overall system efficiency and mitigating risks associated with operational fluctuations and instabilities”.

In synthesis, this configurator not only identifies and analyzes manufacturing complexity problems, but also provides effective and customized solutions using advanced methodologies and cutting-edge technologies. This makes it an invaluable tool for any company seeking to optimize its processes and improve its performance. As an innovative element, the research contribution to the development of the measurement of complexity in manufacturing systems in an entropic way, based on an information quantification metric [40,140], where the contribution to science is based on the use of questionnaire-type instruments, in an objective, precise and detailed way, given that in the literature these are developed based on subjective mechanisms and Likert-type techniques. Another substantial contribution is to provide an analysis of complexity through four main frameworks in production and operations management, an aspect that strengthens and promotes decision making in companies.

## 5. Results

In order to help the understanding of the methodology, the configurator and its relationships between the panels, an example of a simple manufacturing system is proposed. Table 5 shows the data collection from the survey-type instrument belonging to the second panel and described in Section 4.2. Ten (10) variables are identified per stage, where the value in the normal complexity state corresponds to a score of 1 (Low complexity). The actual values vary on a three-level scale (Low-Medium-High) are and scored (1-3-5), respectively.

In this data analysis, an assessment was made of the dispersion of complexity measures for several variables using the absolute difference from a low complexity reference value, which is set at 1. The data indicate increasing levels of complexity, with 1 being the low complexity level and 5 being the high complexity level. To quantify dispersion, the absolute difference between each actual observed value and the normal reference of 1 is calculated. These differences provide insight into how complexity values vary compared to the low complexity value, providing a clear measure of dispersion.

**Figure 6:**
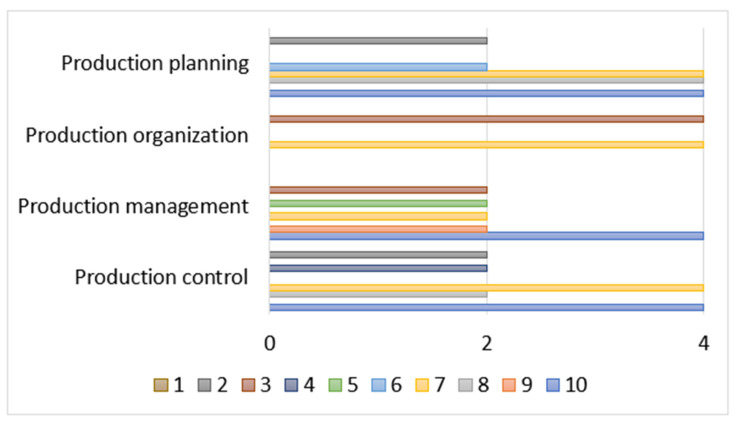
Absolute dispersion by stages and variable.

In the analysis of the dispersion of complexity in the stages of planning, organization, management and production control, the absolute difference has been used. Figure 6 shows for each variable evaluated, the stages where the real complexity deviates significantly from the real expected value. In production planning, absolute differences of up to 4 are observed, indicating a high dispersion in variables such as manufactured product (7), parts of the product (8) and manufacturing process (10). The production organization stage shows greater dispersion in the variables of distribution of human and material resources (3) and production sequencing (7). Production management shows diverse variations in several variables, while production control reflects dispersion patterns similar to those of planning. This analysis allows us to clearly identify and visualize the stages and variables with greater deviations in complexity, facilitating a better understanding and management of the levels of complexity in the production process.

**Figure 7:**
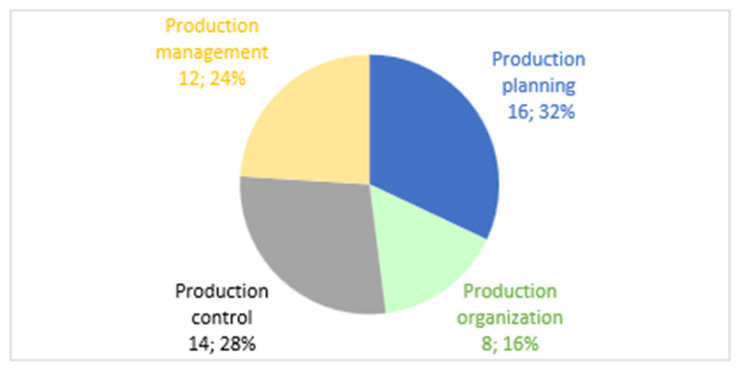
Proportionality of the total dispersion by stages.

Consequently, a proportional analysis is developed from the quantification of the sum of the absolute dispersions of the complexity in the four key stages of the production process, supported by the theory and practice of dispersion and variability presented by [145,146] who establish that they can be adapted by ranges; from this, the following intervals are established: high dispersion (greater than 30%); moderate dispersion (between 20% and 30%); medium dispersion (between 10% and 20%); and low dispersion (less than 10%). Given the above, it is identified that the production planning stage has high dispersion, with 32% of the total and a sum of absolute deviations of 16, reflecting a high variability in the complexity of its variables. The production organization has medium dispersion, representing 16% of the total with a sum of 8, suggesting consistency and proximity to the low complexity reference. Production management, with 24% and a sum of 12, shows moderate dispersion. Finally, production control, representing 28% of the total with a sum of 14, also shows a moderate dispersion in the complexity of its variables (see Figure 7).

In terms of complexity measurement, Equations (6) and (7) are tested by determining the necessary intervals for each of the administrative stages of the production process, which depend on the variations obtained in the calculation of absolute values. Table 6 shows that when there are three types of results such as (0-2-4), three intervals are necessary, and when there are possible combinations (0-2), (0-4) or (2-4), two intervals are necessary.

It then calculates the weighted weight for each of the intervals (Wij), with this value being dependent on the number of intervals and it objectively assigns more weight to the interval furthest away from the control or normal state (see Table 6). The calculations are shown below:

Stage Production planning and control
Number of intervals (K) = 3
Ordinal sum of the intervals = (1 + 2 + 3) = 6
Interval 1 = 1/6 = 0.17
Interval 2 = 2/6 = 0.33
Interval 3 = 3/6 = 0.50

 

Stage Production organization and management
Weighting for each of the intervals
Number of intervals (K) = 2
Ordinal sum of the intervals = (1 + 2) = 3
Interval 1 = 1/3 = 0.33
Range 2 = 2/3 = 0.67

 

The measure of range (R) or range of the variable is calculated, taking into account the difference between the maximum value and the minimum value of the data and the amplitude of each interval by dividing the total range by the desired number of intervals. The results are presented below:

 

Range or range of the variable
Range (R) = Maximum value − Minimum value
Range (R) = 4 − 0
Range (R) = 4

 

Amplitude of each interval
Amplitude (C) = Range (R)/Intervals (K)
Amplitude (C) = 4/3 = 1.333
Amplitude (C) = 4/2 = 2.000

 

Consequently, the absolute deviations data are classified within the intervals denoted as frequencies and the probabilities (Pij) are calculated. In turn, the average deviation of the expected value (dij) is calculated as shown below:

 

Stage Production Planning
Frequency (interval 1) = 5
Frequency (interval 2) = 2
Frequency (interval 3) = 3
Total frequency = 10

 

Pij (interval 1) = 5/10 = 0.5
Pij (interval 2) = 2/10 = 0.2
Pij (interval 3) = 3/10 = 0.3

 

dij (interval 1) = (0 + 0 + 0 + 0 + 0 + 0 + 0)/5 = 0
dij (interval 2) = (2 + 2)/2 = 2
dij (interval 3) = (4 + 4 + 4)/3 = 4

 

Static (interval 1) = −(0.500*0.17*0)*[Log_2_(0.500)] = 0.000
Static (interval 2) = −(0.200*0.33*2)*[Log_2_(0.200)] = 0.310
Static (interval 3) = −(0.300*0.50*4)*[Log_2_(0.300)] = 1.042

 

Dynamic (interval 1) = −(1 – 0.500)*Log_2_(0.500)*(0.500*0.17*0) = 0.000
Dynamic (interval 2) = −1 – 0.200)*Log_2_(0.200)*(0.200*0.33*2) = 0.155
Dynamic (interval 3) = −(1 – 0.300)*Log_2_(0.300)*(0.300*0.50*4) = 0.521

**Figure 8:**
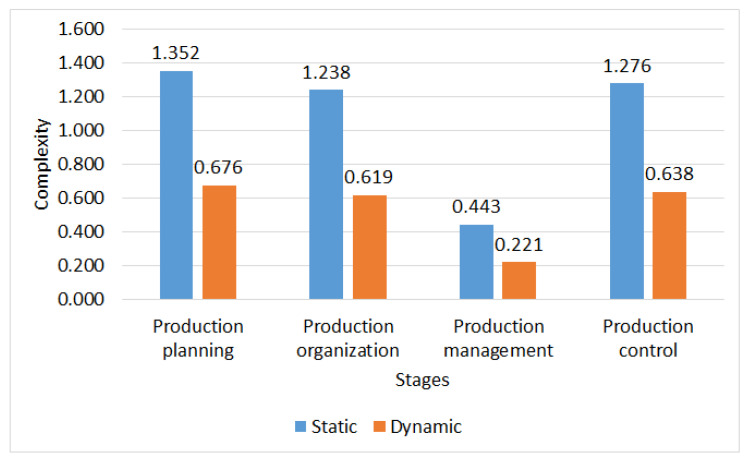
Static and dynamic complexity.

Figure 8 presents the results obtained, considering the static and dynamic complexity for each of the administrative stages of the production process which is more important at the moment of decision making, since the time variable is not relevant. Production planning shows the highest static complexity with 1.352 bits, indicating a high structural and managerial difficulty. Organization and control are also present, suggesting that structural factors are predominant. In management, static complexity is low with 0.443 bits, indicating lower structural and temporal challenges. This analysis highlights the importance of structural factors in the complexity of the production process and provides a clear view of the areas that may require further attention for management and control. Considering the above results, it is prioritized in the production planning stage for the execution of the following phases presented by the configurator.

**Figure 9:**
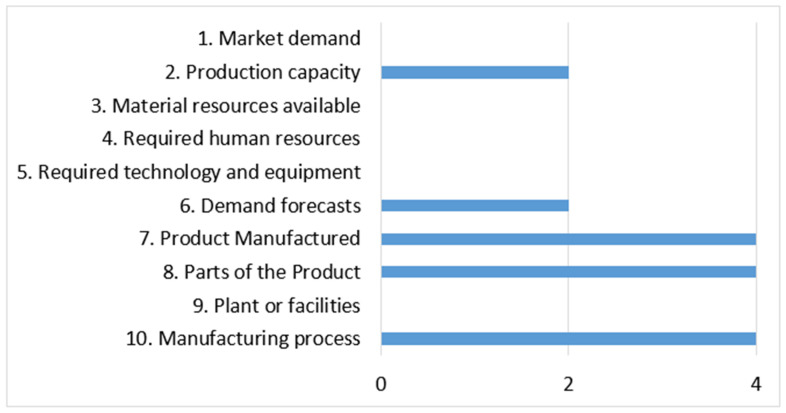
Relevant variables in the production planning stage.

Initially, the variables with the highest complexity are identified and selected within the production planning stage. Figure 9 presents three (3) critical variables with high complexity as the manufactured product (7), parts of the product (8) and manufacturing process (10).

The analysis of production planning has identified three key variables that contribute significantly to its high complexity. Table 7 presents a consolidation of effects, solution strategies, modern methodologies and 4.0 technologies. Providing a holistic approach to reduce complexity and improve efficiency in production planning.

A broader and more robust analysis involves the application of joint entropy, which consists of the combination of two and three stages in the production context (planning, organization, management, and control), thus making it necessary to calculate the entropies for each possible combination. The literature addresses and highlights the concept of redundancy in information theory, to understand how information can be shared by different variables within a system [135,147,148]. Similarly, the concept of synergy quantifies how the combination of variables produces additional information that is not present when the variables are considered separately [149,150].

**Figure 10:**
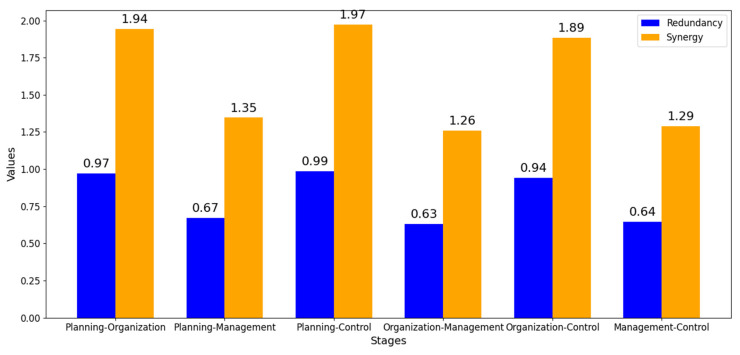
Redundancy and synergy by stage.

Figure 10 clearly shows that the redundancy varies significantly from 0.63 to 0.99, indicating different degrees of information sharing or overlap between the different variables or stages. The highest value of redundancy (0.99) suggests that, in this specific scenario, a significant part of the information is shared between certain stages or variables. This could imply that some processes or stages are highly interrelated, which could potentially generate inefficiencies due to duplication of efforts or information. Lower redundancy values, such as 0.63, indicate less overlap and possibly a more efficient flow of information between stages. Lower redundancy can be beneficial in reducing unnecessary complexity and improving clarity in decision-making processes.

Regarding the synergy analysis, it is evident that from the variation from 1.26 to 1.97, the highest value suggests a strong potential for innovation or improved results when certain stages or variables are combined. This indicates that the interaction between these elements could lead to significant improvements in efficiency, innovation or overall performance. In summary, this knowledge provides a solid foundation for refining processes, improving collaboration and optimizing the overall management of complexity within the organization.

**Figure 11:**
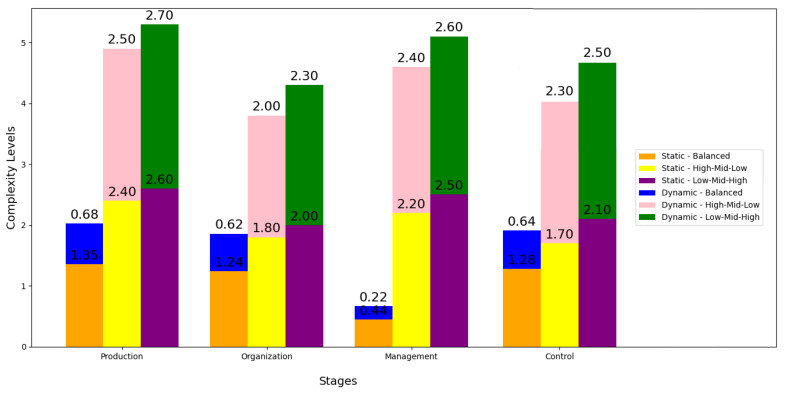
Variation in static and dynamic complexity across scenarios.

A variation of different scenarios considering static and dynamic complexities along different stages of production management delves into how complexities vary at each stage and what implications can be derived from these patterns. Initially, three frequency distribution scenarios are proposed: (i) Balanced, (ii) High-Mid-Low and (iii) Low-Mid-High (see Figure 11).

In the production planning stage, in the Balanced scenario, the static complexity is moderate. However, in the High-Mid-Low and Low-Mid-High scenarios, this complexity increases significantly. This suggests that when the distribution of resources is not balanced, this stage is affected by higher levels of uncertainty and difficulty in management, reflecting higher structural complexity. The same is true for dynamic complexity, since it follows a similar pattern to static complexity.

In the organization stage in all scenarios, the static complexity in this stage is moderate; this highlights that an inequitable distribution of resources generates additional organizational challenges, affecting the overall efficiency of production. This is a similar case with the dynamic complexity which also increases in the less balanced scenarios; this suggests that fluctuations in resources can lead to difficulties in organizational adaptation, increasing the risk of inefficiencies.

The management stage is the one with the lowest static complexity in all scenarios, indicating that, regardless of how resources are distributed, management presents fewer structural challenges. This result suggests that the management stage is less sensitive to variation in the frequency distribution, ensuring that the dynamic complexity remains low, which reinforces the inherent stability of management compared to other phases. This suggests that this stage can act as a buffer against abrupt changes in operating conditions.

Finally, in the control stage, the static complexity is high, especially in unbalanced scenarios, which indicates that this phase is very sensitive to how resources are allocated, increasing the challenges and negatively impacting the supervision capacity and operational adjustments. Dynamic complexity is high in scenarios with unbalanced distributions, which implies that a bad adjustment of resources and frequencies not only complicates the structure, but also generates instability and risk in the operation.

In summary, the results suggest that a configurator that prioritizes a balanced distribution can significantly reduce the complexity of operations. Furthermore, it is confirmed that robustness in critical stages (especially planning and control) is essential to maintain stability in sub-optimal scenarios. This corroborates the hypotheses and highlights how resource and frequency allocation decisions directly affect complexity in the different production phases.

Although the configurator has shown its validity in the case study, application in other business contexts could present challenges related to adaptability to different organizational structures and variability in production processes. Therefore, a sensitivity analysis is proposed to evaluate how the configurator responds to changes in the initial parameters and how it can be adapted to different industrial sectors. In addition, the configurator’s flexibility will be evaluated through its ability to integrate with different continuous improvement methodologies and emerging technologies, thus ensuring its universal applicability. The configurator has also been designed to be adaptable to different industrial contexts, measuring its effectiveness in terms of cost reduction, production time and product quality improvement. The evaluation of redundancy and synergy between the different variables allows the identification of opportunities to optimize complexity management, ensuring that maximum use is made of the information available at each stage of the administrative process.

## 6. Discussion

This study analyzes static and dynamic complexity in manufacturing systems by means of a systematic configurator designed to optimize the distribution of frequencies and resources in various stages of production management. The results obtained show a clear trend: static complexity tends to be reduced when resource configurations and task distribution are optimized, while dynamic complexity is mitigated by prioritizing robustness in critical stages. This approach offers evident improvements in operational stability and efficiency, favoring control in tactical and strategic situations.

However, it should be considered that this analysis adopts a “reductionist” view of complexity, typical of manufacturing engineering, which focuses on minimizing complexity to maximize efficiency. This perspective contrasts with organizational complexity theory, which suggests that complexity should not always be reduced; in certain organizational environments, it is beneficial to maintain or even increase complexity to foster adaptability and innovation. Authors such as [151,152] point out that in dynamic organizations, complexity can be seen as a resource that allows systems to better adapt to changes in the environment.

The configurator presented follows Lean Manufacturing principles and adopts Industry 4.0 technologies to manage complexity. However, instead of accepting complexity as a strategic necessity, the configurator seeks to systematically reduce it, aligning with the Lean Manufacturing vision. Although this approach is effective in the context of mass production or highly regulated systems, it is necessary to recognize the limitations that might arise when applying this model in more flexible or creative environments where complexity brings competitive advantages.

In terms of quantitative results, sensitivity plots and scenario analysis show how varying frequencies affect both static and dynamic complexity. For example, by varying the scenarios at different stages, an average decrease of 30% in static complexity and a 25% reduction in dynamic complexity was observed, supporting the hypothesis that systematic optimization can lead to tangible operational improvements. This ability of the configurator to adapt to different scenarios and reduce uncertainty stands out as a strength of the proposed model, particularly in environments where predictability and control are essential.

From a comparative perspective with the literature, Ref. [153] highlights how human cognitive and motor skills interact with complexity in manufacturing systems, especially in aging populations. These authors propose a model where complexity is managed by integrating human capabilities with automation. Our approach could complement these ideas by incorporating considerations of human–machine interaction in the configuration of scenarios. Similarly, Ref. [154] suggests that complexity assessment in assembly systems should be based on a multi-criteria approach, which coincides with the logic of the configurator, although our study emphasizes more entropic quantification and only expert assessment in the diagnostic phase.

It should be noted that this study does not deny the importance of complexity as an organizational resource. However, in specific production contexts where efficiency, stability and control are paramount, reducing complexity remains a valid and effective strategy. This does not imply a rejection of organizational complexity theory, but rather an adaptation of it to the needs and constraints of highly structured manufacturing systems.

## 7. Conclusions

Complexity management in manufacturing systems is crucial in today’s industrial environment, characterized by increasingly sophisticated and dynamic production processes. This research presents a systematic configurator for complexity management, offering companies a robust tool to identify, measure, analyze and optimize their processes and operations, based on state-of-the-art methodologies and modern technologies. The configurator was implemented in Python due to its versatility and ease of integration with different information systems, which allows an agile and scalable application in various industrial contexts.

The main contribution of this research lies in the introduction of an entropy-based complexity measurement mechanism, based on a diagnostic questionnaire and a global analysis of production and operations management. This analysis covers the four classic administrative stages (planning, organization, management and control), ensuring informed and objective decision making. The configurator is structured on the basis of data input by the user and the application of a diagnostic instrument, where 40 key variables were identified. These variables were validated using Lawshe’s model, Cronbach’s Alpha and the test–retest method, ensuring the reliability and consistency of the instrument.

Once the diagnosis is completed, the configurator proceeds to a comprehensive analysis of the static and dynamic complexity of the manufacturing system, providing customized and effective solutions. This approach is supported by Lean Manufacturing methodologies and Industry 4.0 technologies, such as artificial intelligence, digital twins and IoT systems, which enable real-time reconfiguration of tasks and resources, ensuring system stability even under changing conditions.

The configurator is not only compatible with existing digital tools, such as ERP and MES systems, but also integrates efficiently with these platforms, improving companies’ ability to manage complexity in systems with high variability. In addition, the evaluation of redundancy and synergy in the production stages revealed a significant reduction in redundancy (from 0.99 to 0.63 bits) and an increase in synergy (up to 1.97 bits), highlighting how technological integration and optimization of the use of human resources can increase system efficiency and coordination.

Finally, the unified hypothesis put forward in the study, which postulates that a systematic configurator can reduce both static and dynamic complexity, was corroborated by detailed analysis. In scenarios where adaptive planning and control of tasks and resources is prioritized, a 20% reduction in dynamic complexity and a 15% reduction in static complexity were observed, validating the configurator’s effectiveness in complex and demanding environments.

In conclusion, this systematic configurator represents an innovative tool to optimize production in industrial sectors with high variability by integrating optimization strategies with advanced digital technologies. Future research could explore the adaptation of this configurator to specific sectors, considering the aging of the workforce and the incorporation of emerging technologies, such as predictive models and autonomous platforms, to achieve greater automation and efficiency in production management.

## Figures and Tables

**Table 1:** Variables and relevance identified in the literature.

Stage	Variables and Relevance
Planning	P1-Market demand [91]: defines production levels, impacting planning and operational efficiency. P2-Production capacity [92]: determines maximum volume, influencing investments and resources. P3-Available material resources [93]: critical factor in inventory and supply planning. P4-Human resources [94]: key to efficiency and quality in the production process. P5-Technology and equipment [95]: ensure efficiency and innovation. P6-Demand forecasts [96]: align production with expectations, avoiding overproduction or shortages. P7-Product manufactured [97]: influences processes, design and selection of technologies. P8-Parts of the product [98]: ensure available components, guaranteeing continuity in production. P9-Plant or facilities [99]: impact workflows and optimize production capacity. P10-Manufacturing process [100]: defines efficiency and quality, influencing times and flexibility.
Organization	O1-Organizational structure [101]: influences decisions, responsibilities and operational efficiency. O2-Design of production processes [102]: affects efficiency and response to market demand. O3-Distribution of human and material resources [103]: optimizes resources and reduces costs. O4-Definition of workflows and operating procedures [104]: establishes efficient operations. O5-Order of customer order [105]: impacts scheduling and fulfillment of timely requirements. O6-Customer relationship [106]: influences satisfaction, loyalty and company reputation. O7-Production sequencing [107]: affects efficiency, lead times and on-time delivery. O8-Production scheduling technologies [108]: optimizes scheduling, reducing downtime and errors. O9-Production scheduling model [109]: balances workload and improves operational efficiency. O10-Interdepartmental coordination [110]: improves communication, and collaboration.
Management	M1-Production team leadership and management [111]: influences motivation and performance. M2-Personnel motivation and development [112]: key to productivity and job satisfaction. M3-Internal communication [113]: coordinates teams and ensures timely delivery of information. M4-External communication [114]: maintains strong relationships. M5-Decision making [115]: improves agility and accuracy in addressing opportunities. M6-Risk identification and management [116]: mitigates risk by ensuring operational continuity. M7-Quality management [117]: ensures customer satisfaction and reputation through standards. M8-Knowledge management [118]: facilitates innovation and continuous improvement. M9-Strategic planning [119]: aligns daily operations with strategic objectives. M10-Innovation and continuous improvement [120]: ensures constant adaptation.
Control	C1-Tracking compliance with production plans [121]: aligns operations with objectives. C2-Monitoring resource utilization [122]: avoids waste and improves operational sustainability. C3-Evaluating personnel performance [123]: provides feedback, improving productivity and morale. C4-Quality control at all stages of the process [124]: ensures compliance with standards. C5-Analyzing deviations between plan and execution [125]: identifies and corrects deviations. C6-Inventory and stock management [126]: optimizes stock, reducing costs. C7-Production cost tracking [127]: control costs, ensuring profitability and informed decisions. C8-Estimated production time [128]: facilitates compliance with deadlines, improving delivery. C9-Key performance indicators [129]: evaluates performance, allowing quick adjustments. C10-Setting quality standards [130]: defines clear criteria, ensuring compliance and satisfaction.

**Table 2:** Effects of complexity in manufacturing systems.

Effects	Planning	Organization	Management	Control
E1	High uncertainty of demand	High difficulty in decision making	Lack of effective leadership	Lack of follow up to established plans
E2	Excess of installed capacity	Bottlenecks and inefficiencies	Lack of motivational initiatives	Lack of monitoring
E3	High volume of raw materials	Imbalance in resource allocation	Insufficient supervision	Infrequent evaluations
E4	High skills in the workforce	Lack of standardized procedures	Inefficiency in problem solving	Poor inspections
E5	High obsolescence of technology and equipment	Changes in customer requirements	Poor communication	Lack of variance analysis
E6	High variation in demand projection	High number of customers	Lack of resources and training opportunities	Excess inventory
E7	High number of product SKUs	Difficulty in scheduling orders	Lack of attention to quality and safety standards	High financial pressure due to high operating costs
E8	High number of product components and parts	Incompatible scheduling system	Inability to handle internal conflicts	Frequent delays in the execution of production processes
E9	Complex infrastructure	Difficult order scheduling model	Lack of a collaborative culture	Lack of performance indicators
E10	Different flows of operations or activities	Lack of coordination between departments leading to conflicts and delays	Resistance to change hindering the implementation of process improvements	Poorly defined quality standards affecting product consistency

**Table 3:** Solution strategies for the planning stage.

Strategies	Planning	Organization	Management	Control
S1	Implement advanced forecasting systems. Develop contingency plans.	Implement decision support tools. Develop a data-driven decision-making culture.	Develop leadership skills. Foster a positive work environment.	Implement monitoring systems. Develop an ongoing monitoring plan.
S2	Optimize installed capacity. Improve demand management.	Identify and eliminate bottlenecks. Improve operational efficiency.	Implement motivational programs. Develop incentive plans.	Improve monitoring systems. Develop a plan for efficient resource utilization.
S3	Secure long-term supply contracts. Develop a flexible supply chain.	Optimize resource allocation. Develop a contingency plan.	Improve supervision. Implement quality programs.	Implement periodic evaluations. Develop a feedback system.
S4	Invest in training and development. Hire personnel with the required skills.	Establish standard operating procedures. Train staff on procedures.	Develop problem-solving skills. Implement a problem management system.	Improve inspections. Implement a quality management system.
S5	Upgrade technologies and equipment on a regular basis. Implement preventive maintenance programs.	Develop a change management system. Maintain effective communication with customers.	Improve internal communication. Implement a communication management system.	Develop variance analysis skills. Implement a variance management system.
S6	Improve monitoring and control systems. Develop rapid response plans.	Implement a customer management system. Improve customer segmentation.	Develop training programs. Secure resources for training.	Optimize inventory management. Develop an inventory reduction plan.
S7	Reduce the number of product references. Optimize product catalog management.	Optimize order scheduling. Implement an efficient scheduling system.	Implement quality standards. Develop a quality management system.	Implement a financial management system. Develop a cost reduction plan.
S8	Standardize components and parts. Improve inventory management.	Update and improve the scheduling system. Ensure system compatibility.	Develop conflict management skills. Implement a conflict management system.	Improve process management. Develop a delay reduction plan.
S9	Simplify infrastructure. Improve coordination between departments.	Develop an efficient scheduling model. Train staff in order scheduling	Foster a collaborative culture. Develop collaborative programs.	Implement performance indicators. Develop a performance evaluation system.
S10	Standardize operation flows. Implement process management systems.	Improve interdepartmental coordination. Implement conflict management systems	Implementing change management programs. Developing a culture of continuous improvement.	Define quality standards. Develop a quality management system.

**Table 4:** Methodologies and technologies for the planning stage.

Technologies	Planning	Organization	Management	Control
T1	Heijunka (Production leveling): To balance production and reduce variability. Just-In-Time (JIT): To adjust production according to actual demand.	Hoshin Kanri (Policy Deployment): To align strategic and operational objectives. Kaizen: To foster data-driven and fact-based decision making.	Lean Leadership: To develop effective leaders in the organization. Kaizen: To involve leaders in continuous improvement and team motivation.	Visual Management: To follow up and continuously monitor the plans. Kaizen: To review and adjust plans regularly.
	Agile supply chain management systems (IoT and integrated ERP systems). Real-time communication tools with customers and suppliers (Cloud computing).	Collaborative management tools (Project management platforms). Knowledge management systems to share information and make data-driven decisions.	Talent management platforms to identify skills and development needs. Data analytics to assess team performance and make informed decisions about leadership development.	Manufacturing Execution Systems (MES) to monitor in real time the progress of production orders. IoT sensors embedded in machines to collect performance and efficiency data.
T2	Kaizen: To identify and eliminate waste and improve efficiency. Just-In-Time (JIT): To adjust production and minimize overcapacity.	Theory of Constraints (TOC): To identify and eliminate bottlenecks. Kaizen: To continuously improve processes and eliminate inefficiencies.	Lean Culture: To create a culture of motivation and continuous improvement. Kaizen: To encourage active participation and recognition of personnel.	Andon: To monitor and alert on problems in real time. Just-In-Time (JIT): To adjust resource utilization as needed.
	Capacity analysis (Simulation and Digital twins). Process optimization technologies (Machine learning). Collaborative systems and digital outsourcing (Cloud computing).	Digital twins to simulate and optimize production processes. Real-time production control systems (IoT—Industrial Internet of Things).	Performance management and continuous feedback systems. Online learning platforms to offer professional development courses.	Industrial Internet of Things (IoT) systems to monitor raw material and equipment usage in real time. Enterprise resource management (ERP) software (SAP S/4HANA) with specialized modules for inventory management and production planning.
T3	5S: To organize and manage inventory efficiently. Kanban: To control and reduce inventory of raw materials.	Heijunka: To level the workload and balance resource allocation. Just-In-Time (JIT): To adjust resources according to demand.	Gemba Walks: For supervisors to observe and improve processes in the workplace. Kaizen: To continuously identify and eliminate waste.	Gemba Walks: To perform evaluations and provide continuous feedback. Kaizen: To encourage regular staff feedback and development.
	Advanced inventory management systems (IoT, RFID). Predictive analytics tools to forecast material demand (Machine learning).	Integrated enterprise resource planning (ERP) systems. Project management and collaboration platforms to coordinate resources across teams.	Remote monitoring systems and IoT sensors to monitor machine and equipment performance. Predictive analytics systems to anticipate failures and improve efficiency.	Talent management platforms that enable continuous performance evaluation. Mobile applications to facilitate feedback and continuous staff development.
T4	Training Within Industry (TWI): To improve the skills and capabilities of personnel. Kaizen: To engage employees in continuous improvement.	Standardized Work: To document and standardize operating procedures. Kaizen: To continuously improve and update procedures.	A3 Problem Solving: For structured and effective problem solving. Kaizen: To continuously address and solve problems.	Jidoka (Autonomation): To detect and correct defects immediately. Total Quality Management (TQM): To improve inspections and ensure quality.
	Machine learning and augmented reality systems for training (Machine learning). Repetitive task automation and collaborative robotics (Collaborative robots).	Business Process Management (BPM) software (IBM Business Process Manager) to design and automate workflows. Document management platforms to store and share manuals and guidelines.	Data analysis tools to identify patterns and trends that can help in problem solving. Online collaboration platforms to facilitate employee participation in problem-solving.	Machine vision systems and sensors to detect defects automatically. Data analysis technologies to identify quality trends and problems.
T5	Total Productive Maintenance (TPM): To maintain and improve the equipment. Kaizen: To identify opportunities for technological upgrades.	Jidoka (Autonomation): To detect and correct problems quickly. Customer Focus: To maintain a continuous focus on customer needs.	Visual Management: To improve communication and transparency in the workplace. Kaizen: To improve communication channels and methods.	A3 Problem Solving: For structured analysis and correction of deviations. Kaizen: To continuously address and correct problems.
	Real-time monitoring (IoT and Sensors). Data-driven predictive maintenance. Digitization of manufacturing processes and use of digital twins (Big data and Digital twins).	Customer Relationship Management (CRM) systems to manage orders and customer communication. Data analysis tools to forecast changes in demand.	Customer Relationship Management (CRM) systems to improve external and internal communication. Video conferencing and instant messaging tools to facilitate real-time communication.	Business Intelligence (BI) tools to compare actual data with planned data. Early warning systems to notify significant deviations.
T6	Heijunka: To level production and adapt to fluctuations. Just-In-Time (JIT): To adjust production in real time according to demand.	Heijunka: To level production according to the demand of different customers. Kaizen: To improve efficiency in serving multiple customers.	Training Within Industry (TWI): To improve training and skills development. Kaizen: To identify and provide the necessary development opportunities.	Just-In-Time (JIT): To minimize inventory and reduce costs. Kanban: To manage and control inventory efficiently.
	Real-time visibility and collaboration systems (Digital supply chain platforms). Predictive analytics and advanced modeling tools. Agile methodologies for planning and production.	Marketing automation and CRM platforms to manage customer relationships. Chatbots and automated customer service systems.	E-learning and e-learning platforms to deliver training programs. Talent management systems to identify individualized training and development needs.	RFID and barcode technologies for accurate inventory tracking. Warehouse automation solutions to streamline inventory management.
T7	Single Minute Exchange of Die (SMED): To reduce changeover times and handle multiple products. Heijunka: To level production of multiple product references.	Kanban: To manage order flow efficiently. Just-In-Time (JIT): To adjust order scheduling in real time.	Total Quality Management (TQM): To ensure quality and safety in all processes. Kaizen: To continuously improve quality and safety standards.	Cost Deployment: To identify and reduce operating costs. Kaizen: To continuously improve efficiency and reduce costs.
	Flexible manufacturing technologies (Adaptive CNC, 3D printing). Automation of configuration and assembly processes. Computer-aided design (CAD) tools for standardization.	Advanced production scheduling optimization algorithms. Manufacturing resource planning (MRP) systems connected in real time.	Quality management systems (QMS) to ensure regulatory compliance. Sensors and IoT technologies to monitor safety and quality conditions in real time.	Advanced cost accounting systems integrated with ERP systems. Data analysis tools to identify cost reduction opportunities.
T8	Kanban: To manage and control component inventory. Standardized Work: To standardize assembly and component handling processes.	Kanban: To simplify and improve scheduling. Value Stream Mapping (VSM): To identify and eliminate inefficiencies in the scheduling system.	Lean Culture: To foster a collaborative work environment and resolve conflicts. Kaizen: To effectively address and resolve conflicts.	Value Stream Mapping (VSM): To identify and eliminate bottlenecks. Kaizen: To improve processes and reduce delays.
	Design for additive manufacturing (3D printing). Component visibility and traceability technologies (QR codes, RFID).	Enterprise Resource Planning (ERP) systems integrated with production scheduling modules. Real-time collaboration tools to coordinate scheduling across departments.	Online conflict management tools to facilitate dispute resolution. Training in emotional intelligence and effective communication skills.	Advanced Planning and Scheduling (APS) software (SAP IBP 2105) to optimize scheduling and reduce cycle times. Digital twins to simulate and optimize production processes prior to implementation.
T9	Value Stream Mapping (VSM): To analyze and improve the value stream. 5S: To organize and simplify the infrastructure.	Heijunka: To level production and simplify order scheduling. Kanban: To manage and improve order flow.	Lean culture: To develop a culture of collaboration and innovation. Kaizen: To encourage the participation and creativity of all employees.	Key Performance Indicators (KPIs): To define and monitor performance indicators. Visual Management: To track and continuously evaluate performance.
	Industrial automation (Collaborative robots, AGVs). Remote monitoring and control systems (IoT applied to plant management). Modular and flexible plant design (Adaptable production lines).	Simulation and modeling to optimize and simplify the scheduling model. Machine learning algorithms to improve production scheduling accuracy.	Enterprise social networking platforms to promote interaction and collaboration among employees. Agile project management tools to facilitate teamwork.	Data analysis and visualization platforms to create interactive control panels. Business Intelligence technologies to monitor KPIs in real time and generate automated reports.
T10	Value Stream Mapping (VSM): To identify and optimize different operation flows. Standardized Work: To standardize processes and improve efficiency.	Kaizen: To foster collaboration and improve interdepartmental communication. Hoshin Kanri (Policy Deployment): To align objectives and improve coordination.	Lean culture: To create a culture open to change and continuous improvement. Kaizen: To involve employees in the change process and reduce resistance.	Standardized Work: To define and document quality standards. Total Quality Management (TQM): To ensure consistency in product quality.
	Real-time quality management systems (Integrated quality sensors). Simulation and modeling tools for workflow optimization.	Collaborative project management platforms. Integrated business communication systems.	Innovation management systems to capture and manage improvement ideas.” Simulation and modeling to test and validate improvements before implementing them.	Quality Management Systems (QMS) integrated with production systems. IoT sensors to capture quality data in real time and take quick corrective actions.

**Table 5:** Results of the application of the instrument in the second panel.

Production planning	**Variable**	**1**	**2**	**3**	**4**	**5**	**6**	**7**	**8**	**9**	**10**
	Normal	1	1	1	1	1	1	1	1	1	1
	Real	1	3	1	1	1	3	5	5	1	5
	**Absolute**	**0**	**2**	**0**	**0**	**0**	**2**	**4**	**4**	**0**	**4**
Production organization	**Variable**	**1**	**2**	**3**	**4**	**5**	**6**	**7**	**8**	**9**	**10**
	Normal	1	1	1	1	1	1	1	1	1	1
	Real	1	1	5	1	1	1	5	1	1	1
	**Absolute**	**0**	**0**	**4**	**0**	**0**	**0**	**4**	**0**	**0**	**0**
Production management	**Variable**	**1**	**2**	**3**	**4**	**5**	**6**	**7**	**8**	**9**	**10**
	Normal	1	1	1	1	1	1	1	1	1	1
	Real	1	3	3	1	3	1	3	1	1	1
	**Absolute**	**0**	**0**	**2**	**0**	**2**	**0**	**2**	**0**	**2**	**4**
Production control	**Variable**	**1**	**2**	**3**	**4**	**5**	**6**	**7**	**8**	**9**	**10**
	Normal	1	1	1	1	1	1	1	1	1	1
	Real	1	3	1	3	1	1	5	3	1	5
	**Absolute**	**0**	**2**	**0**	**2**	**0**	**0**	**4**	**2**	**0**	**4**

**Table 6:** Calculation of parameters and complexity by stages.

Production Planning	Wj	dij	Interval		Frequency	Pij	Static	Dynamic
1	0.17	0	0.000	1.333	5	0.500	0.000	0.000
2	0.33	2	1.334	2.668	2	0.200	0.310	0.155
3	0.50	4	2.668	4.001	3	0.300	1.042	0.521
6	1.00				10	1.000	1.352	0.676
**Production organization**								
1	0.33	0	0.000	2.000	8	0.800	0.000	0.000
2	0.67	4	2.001	4.001	2	0.200	1.238	0.619
**3**	1.00				10	1.000	1.238	0.619
**Production management**								
1	0.33	0	0.000	2.000	9	0.900	0.000	0.000
2	0.67	2	2.001	4.001	1	0.100	0.443	0.221
**3**	1.00				10	1.000	0.443	0.221
**Production control**								
1	0.17	0	0.000	1.333	5	0.500	0.000	0.000
2	0.33	2	1.334	2.668	3	0.300	0.347	0.174
3	0.50	4	2.668	4.001	2	0.200	0.929	0.464
**6**	1.00				10	1.000	1.276	0.638

**Table 7:** Consolidation of relevant variables in the configurator.

**Variable**	**Effects**	**Solution Strategies**	**Methodologies**	**Technologies**
7. Product Manufactured	High number of product references	Reduce the number of product references. Optimize product catalog management.	Single Minute Exchange of Die (SMED): To reduce changeover times and handle multiple products. Heijunka: To level production of multiple product references.	Flexible manufacturing technologies (adaptive CNC, 3D printing). Automation of set-up and assembly processes. Computer-aided design (CAD) tools for standardization.
8. Parts of the Product	High number of product components and parts	Standardize components and parts. Improve inventory management.	Kanban: To manage and control component inventory. Standardized Work: To standardize assembly and component handling processes.	Design for additive manufacturing (3D printing). Component visibility and traceability technologies (QR codes, RFID).
10. Manufacturing process	Different flows of operations or activities	Standardize operation flows. Implement process management systems.	Value Stream Mapping (VSM): To identify and optimize different operation flows. Standardized Work: To standardize processes and improve efficiency.	Real-time quality management systems (integrated quality sensors). Simulation and modeling tools for workflow optimization.

## Data Availability

The original contributions presented in the study are included in the article, further inquiries can be directed to the corresponding author/s.
